# Diseases and Physical Education of Children

**Published:** 1851-01

**Authors:** 


### ARTICLE VI.

*A Treatise on the Diseases and Physical Education of Children.* By JOHN EBERLE, M. D., late Prof. of the Theory and Practice of Medicine in Transylvania University, &c. &c. &c. Fourth edition, with notes and large additions, by THOMAS MITCHELL, A. M., M. D., Prof. of the Theory and Practice of Medicine in the Philadelphia College of Medicine; late Prof. of Materia Medica, and Therapeutics in Transylvania University; lecturer on Obstetrics, and the diseases of women and children, &c. &c. &c., Philadelphia, Lippincott, Grambo & Co, successors to Grigg, Elliot & Co., No. 14 North Fourth St., 1850, pp., 768, 8 vo. (From the publishers. For sale by J. Keen Jr. & Bro., Chicago)

Prof. Mitchell informs us that he had it in contemplation to prepare a new work on the Diseases of Children; but the fact of the publishers having in their possession the stereotye plates of Eberle's work, induced him merely to add a supplement thereto. We think his original design was the best, for we do not think a new edition of Eberle was called for, and the possession of stereotype plates surely was not sufficient reason for its publication. When Eberle's work was published, it was a very fair book, but no physician, who has kept pace with the improvements in this department of Practicpl Medicine, would now recommend it as a guide for students.

In the sequel Dr. Mitchell has given a very interesting and pretty complete summary of the existing condition of this branch of Practical Medicine, although he has allowed some matters to escape his notice, for instance he has made no mention of Dr. Brainard's method of treating Spina Bifida, which we think he might have noticed, and which, to say the least, would have been interesting to his readers.

The book is beautifully printed on very good paper, and is very substantially bound. M.

